# The role of convolutional neural networks in scanning probe microscopy: a review

**DOI:** 10.3762/bjnano.12.66

**Published:** 2021-08-13

**Authors:** Ido Azuri, Irit Rosenhek-Goldian, Neta Regev-Rudzki, Georg Fantner, Sidney R Cohen

**Affiliations:** 1 Weizmann Institute of Science, Department of Life Sciences Core Facilities, Rehovot 76100, Israel; 2 Weizmann Institute of Science, Department of Chemical Research Support, Rehovot 76100, Israel; 3 Weizmann Institute of Science, Department of Biomolecular Sciences, Rehovot 76100, Israel; 4 École Polytechnique Fédérale de Lausanne, Laboratory for Bio- and Nano-Instrumentation, CH1015 Lausanne, Switzerland

**Keywords:** atomic force microscopy (AFM), deep learning, machine learning, neural networks, scanning probe microscopy (SPM)

## Abstract

Progress in computing capabilities has enhanced science in many ways. In recent years, various branches of machine learning have been the key facilitators in forging new paths, ranging from categorizing big data to instrumental control, from materials design through image analysis. Deep learning has the ability to identify abstract characteristics embedded within a data set, subsequently using that association to categorize, identify, and isolate subsets of the data. Scanning probe microscopy measures multimodal surface properties, combining morphology with electronic, mechanical, and other characteristics. In this review, we focus on a subset of deep learning algorithms, that is, convolutional neural networks, and how it is transforming the acquisition and analysis of scanning probe data.

## Review

### Introduction: traditional machine learning vs deep learning

Machine learning is a subfield of artificial intelligence. It was defined by Arthur Samuel of IBM in 1959 as the “Field of study that gives computers the ability to learn without being explicitly programmed” [1]. This broad definition includes a variety of tasks including, but not limited to, classification, regression (prediction of quantitative data values), translation (for instance of languages), anomaly detection, de-noising, clustering (grouping similar objects together), and data generation. In this review our major concern is with images, which are most relevant to certain aspects of machine learning as will be described below. Machine learning is thus an umbrella term for many different types of learning [2–6].

Computer vision, which can be used to derive complex information from digital images and videos (equivalent and even superior to expert humans), frequently relies on machine learning, and image classification is one of the most basic tasks. “Classifying” means assigning the image to a specific category [7], such as dog/cat for kind of animal, healthy/diseased for cells, particular orientation/configuration for an adsorbed molecule. There could be many different classes in a data set: Red blood cells in wide-field images can be classified into ten different classes based on morphological differences [8]. More advanced tasks in computer vision are object localization and detection. “Detection” of objects in an image is usually performed by defining bounding boxes around the objects of interest, outputting the spatial coordinates defining locations and sizes of bounding boxes, while “localization” is a specific case of detection of a single object in an image [9]. An even finer distinction is “segmentation”. This involves dividing an image into several parts, sets of pixels, to locate objects and identify specific structures and boundaries, namely “pixel classification” [7,10]. For example, in medical image diagnosis, the segmentation of organs allows for the quantification of their volume and shape [11]. In many biological applications, nucleus identification (segmentation) is used as a good reference for image analysis approaches, that is, cell counting or tracking, and protein localization [12]. The segmentation can be performed as a “semantic segmentation”, which classifies image pixels into specific categories, while “instance segmentation” additionally segments each object in the image.

When used for image processing, traditional machine learning algorithms analyze images either by flattening the data to 1D vectors, or by extracting features one-by-one and transforming them to 1D vectors. This leads to a loss of information of the neighborhood of a given pixel, and in some cases to an extremely inefficient use of computer resources. Therefore, these image processing techniques require expertise in the specific discipline to identify the important features characterizing the categories, and to then extract them from the images [13–16]. These expert-defined features are fed into the machine learning classification algorithms to create a model [17–23] that learns the best mapping between the features and the different categories. This model is subsequently applied on new, unseen images. The approach in which the model is trained on a set of images that are labeled by their respective categories is termed “supervised learning”. This labeling may in itself be a formidable task, adding to the computational demand. For this reason, techniques have been developed to train on unlabeled images, termed “unsupervised learning” [24]. For completeness, we also mention here a third type of learning, that is, “reinforcement learning”, in which the model learns to independently reach a goal within a given environment by trial and error, reinforced by operator chosen rewards or penalties resulting from the result of these decisions.

Neural networks were first proposed by Warren McCulloch and Walter Pitts in 1943 [25]. This provided the groundwork for the eventual use of artificial neural networks (ANNs) in machine learning. ANNs comprise an end-to-end process, where the neural network learns, extracts, and selects those features most appropriate for the given task. Notwithstanding the terminology, an ANN does not work the same way a biological neuron works, but the fundamental idea of response to input being selectively passed forward in a network is similar [26]. Following the initial work of McCulloch and Pitts [25], the analogy of neural networks to the physiological ones was furthered in the 1962 work of Hubel and Wiesel, which showed that a set of neurons arranged in a column extending inwards from the brain surface all respond to stimuli of a specific orientation and location [27]. For instance, a particular column could fire when a vertical edge is observed. Artificial neural networks are now applied to various tasks in image analysis such as image classification, object detection, image retrieval, and segmentation. A subset of ANNs, deep neural networks (DNNs), uses a set of algorithms in machine learning based on artificial neural networks, which allow for a much more autonomous method for characterizing images than traditional algorithms [26,28–32]. The “architecture” for these networks will be described below. In this review, we adopt the term traditional machine learning when the algorithms applied are not part of the deep learning family of algorithms.

The most widely used neural network type in image analysis is the convolutional neural network (CNN) [26,28–31]. CNN uses several manipulations to reduce the demand on computing resources and increase efficiency, as will be described below. In the relatively short time since their development, deep neural networks have registered remarkable success in image analysis [33]. Image recognition tasks performed using deep architectures can perform better than humans [34–35]. It is useful in many disciplines, such as biology, chemistry, physics, medicine, vehicle industries, security, and food industries [36–67]. The utility of CNNs in image classification lies in the fact that it uses algorithms that are extremely efficient in learning, extracting, and selecting the particular features that give the best mapping between the input images and the given set of categories in order to create a model that can be used on new images. As a result, no prior knowledge in the specific discipline is required and practitioners can solve the given task without being experts in the field.

**Figure 1:**
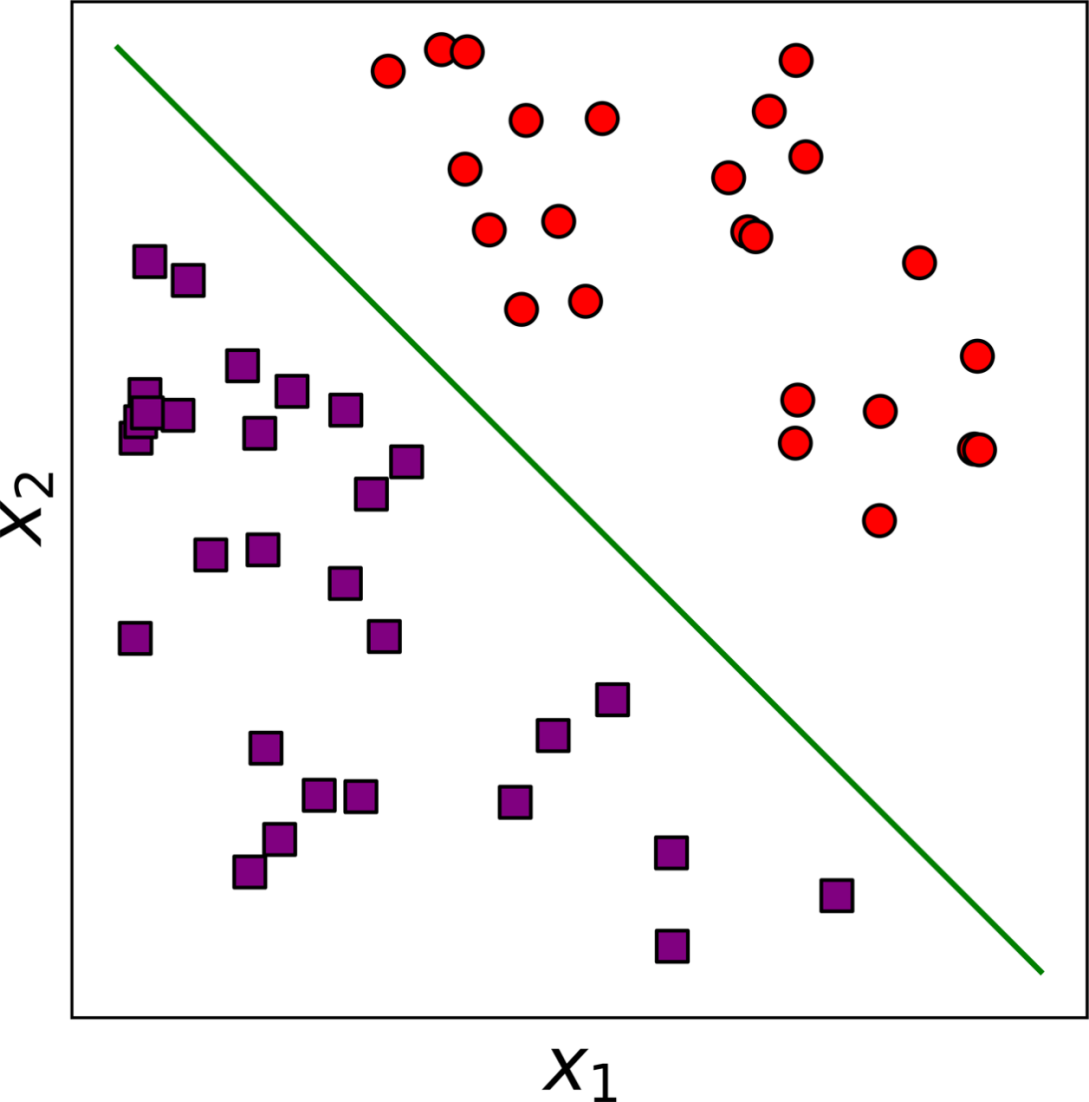
Linear decision boundary (green straight line) that separates between samples belonging to two different categories, that is, filled purple rectangles (associated more with values of features *X*_1_ and *X*_2_ that are to the left of the decision boundary), and filled red circles (associated more with values of features *X*_1_ and *X*_2_ that are to the right of the decision boundary). For instance, if the model is distinguishing between two different domains in an image, *X*_1_ could represent shape and *X*_2_ roughness. Or in medical diagnosis, *X*_1_ and *X*_2_ may be body mass index and blood pressure.

The design of machine learning algorithms enables them to learn their own decision boundaries separating the different categories in a classification task [68–70]. This boundary is defined by the algorithm. A common example is a linear decision boundary, that is, straight lines that can differentiate between samples from the different categories with respect to the given features (Figure 1). Higher-order polynomial degree decision boundaries are also used, but need to be set a priori and are limited only to the specific shape at hand. In contrast, deep learning algorithms learn the optimal decision boundaries directly from the data and in principle can learn any non-linear, complex decision boundary.

**Figure 2:**
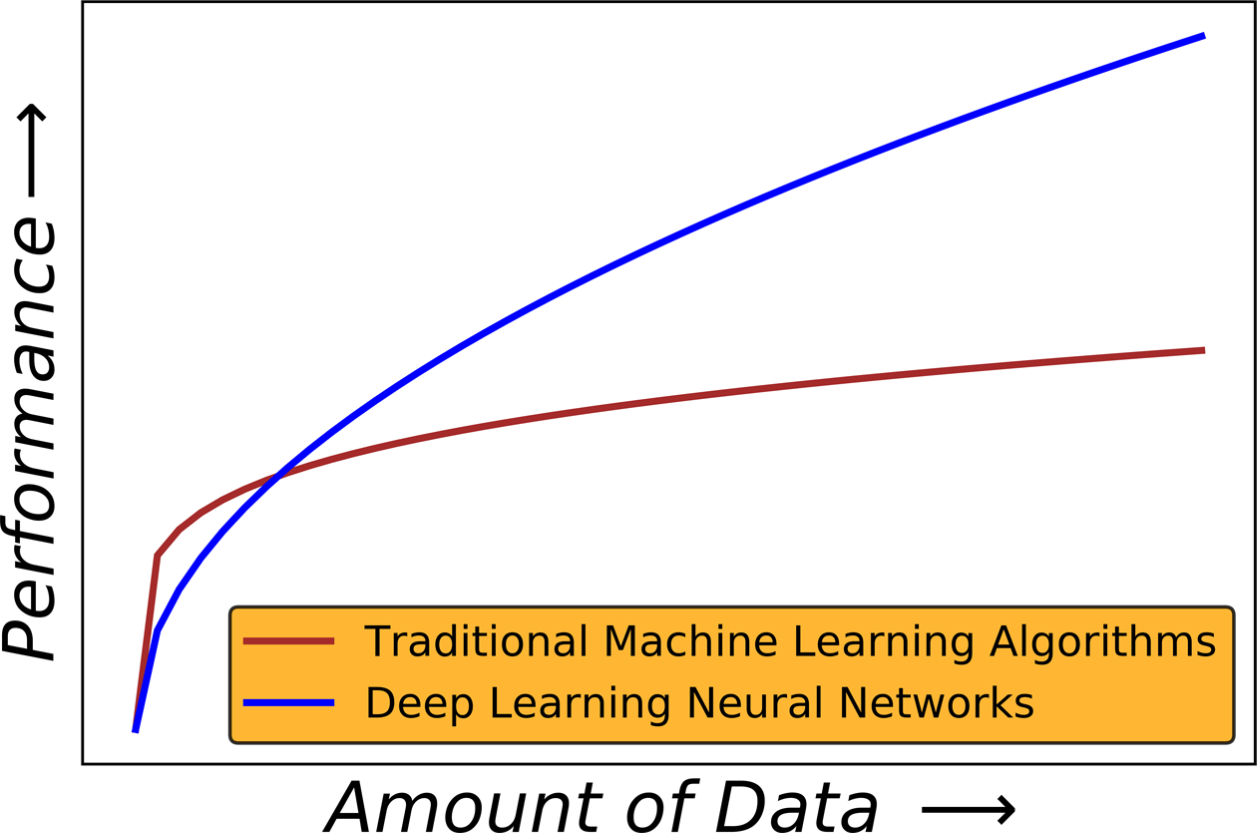
Performance as function of sample data set size for traditional machine learning algorithms and deep learning neural networks.

In general, deep learning works best when the sample data set size is large [71]. In Figure 2, a typical qualitative comparison between performance of traditional machine learning and deep learning algorithms as function of data set size demonstrates the advantage of deep learning over traditional machine learning algorithms for large sample data sets. Since deep learning models have, in general, many more parameters to learn and optimize compared to traditional machine learning algorithms, when the requirement of large sample data set size is added, significant computing resources are needed. The computing developments of the last decade, which enabled the utilization of advanced central processing units (CPUs) in parallel as well as advanced graphical processing units (GPUs) and tensor processing units (TPUs), revolutionized the use of deep learning, and together with the developments in advanced deep learning algorithms have greatly increased the computational speeds.

A ramification of the requirement of large data sets for CNN is that it is best applied where large amounts of data can be generated, either experimentally or computationally. Nonetheless, there are cases where the data set size is small, so that traditional machine learning algorithms are preferred to yield high performance. Their application requires domain expertise (or feature engineering, that is, the process of extracting features from raw data using prior, expert, knowledge to determine what is relevant, as well as designing new complex features from combinations of simpler, fundamental ones). For problems where this is readily done, and data sets are small, the traditional methods may be more appropriate. Nonetheless, CNNs have been successfully applied to specific problems in image analysis with relatively small data sets as will be shown in some examples below.

Machine learning in nanoscience [72] and microscopy [73], including scanning probe microscopy [74], has emerged in the past decade as a catalyst for a better understanding of data and exploitation of the experimental tools we have at hand. The literature referred to above, and in the rest of this manuscript, points to some of the major developments and hopefully captures some of the excitement surrounding them. In this work, after introducing some of the basic structure and functionality of NNs, we concentrate on the applications of deep learning, primarily CNN, to scanning probe microscopy (SPM). The combination of improved scanning speeds, which will enable acquisition of large image data sets, and computational techniques to generate training images forebodes an increasing role of this method for scanning probe techniques. Deep learning applications, of which a few representative case studies are presented here, include both image and spectroscopic SPM data. In general, CNN has proven to be extremely efficient for imaging applications and seems well poised to radically change the way the images are acquired and analyzed.

### Deep learning ANNs

**Figure 3:**
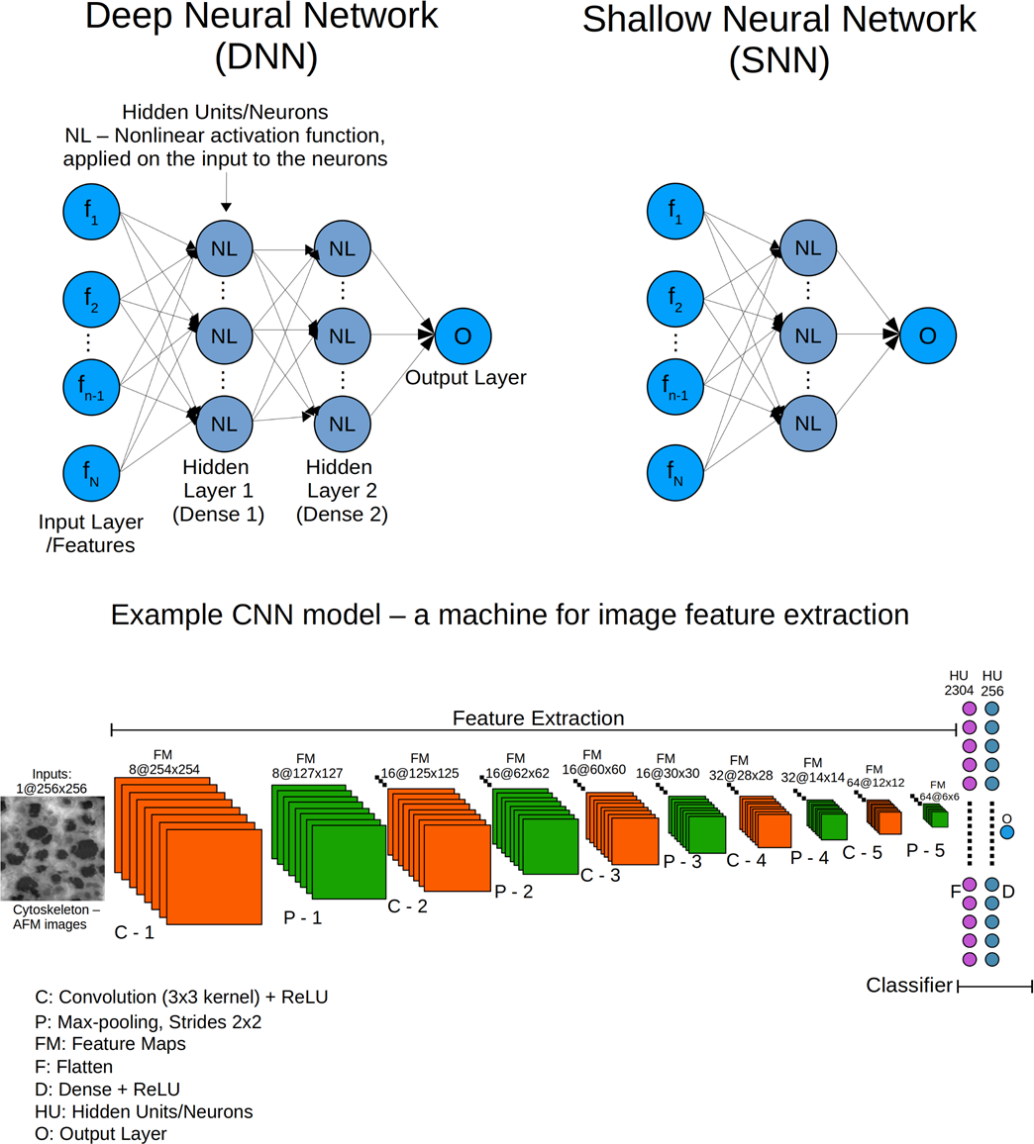
Upper left: example of a deep neural network (DNN). Upper right: example of a shallow neural network (SNN). Bottom: basic components of a CNN model. In this example, a 2D image of 256 × 256 pixels is input to the CNN. C-1 is the first convolution (see “Convolutional layer”) with eight kernels of size 3 × 3 followed by ReLU activation (see “Activation layer”) that yields eight “activated” feature maps of size 254 × 254 pixels. The size of the image drops from 256 × 256 to 254 × 254 due to the convolution operation with a kernel size of 3 × 3. Next is P-1, that is, the max-pooling operation (see “Pooling layer”) on each feature map that is the output of C-1 with a stride size of 2 × 2. The max-pooling operation reduces the size of each “activated” feature map by a factor of two. Convolutional + activation + pooling layers form a block of operations that repeats each time the number of kernels increases or is kept equal to that of the previous block. The output of the last block (here P-5) is flattened (see “Flatten layer”) and fed into the fully connected layer followed by ReLU activation. A few dense layers can be connected sequentially. The last dense layer is connected to the output layer with the appropriate activation to yield the classification category. Adapted from [67], Copyright © Dekel et al., licensed under a Creative Commons Attribution 4.0 International License, http://creativecommons.org/licenses/by/4.0/”.

Deep learning is a class of algorithms in machine learning based on ANNs [26,28–29]. There are several main types, each designed for different tasks. The most basic ANN for the instance of image classification is composed of the input layer, which contains an image set, a hidden layer, which analyzes the images to determine and classify their features, and the output layer, which contains the different categories of the classification task. The hidden layer contains the “neurons” that connect the input layer (features) to the output layer (categories) and learns the relationship between them. This is done by processing their input to decide if, and how the information will be passed forward. The features are fed to each neuron by linear transformations using the NN model parameters, i.e., weights (parameters that associate importance to each feature) and bias (parameters that enable the model to account for patterns that are shifted from the origin which, for instance, would result in upwards or downward shift of the boundary line in Figure 1). Then, a non-linear function is applied for each neuron. Without this step, termed “activation” (see below), multiple layers add no new knowledge to the problem. This non-linear function enables the neural network to learn complex non-linear decision boundaries instead of the linear decision boundaries, and prevents it from collapsing to a neural network without hidden layers. Application of this linear decision boundary has no benefit over logistic/linear regression, which is used for classification/regression in supervised machine learning. The weights and biases are optimized by the backward propagation algorithm (see loss function section) to yield the decision boundaries that best distinguish between samples belonging to different categories. By definition, a network containing only one hidden layer is called a shallow neural network (SNN) (Figure 3, upper right), and one containing more than one hidden layer is called a deep neural network (DNN) (Figure 3, upper left [75]). These ANNs are also called fully connected ANNs and were the first, basic neural networks developed. In general, the first hidden layer learns low-level features and as it becomes deeper, higher-level and more complex features are revealed. The neural network learns the higher-level features from characteristic relations between the lower-level ones. They are used to develop more sophisticated and complex decision boundaries and have been applied successfully in many disciplines such as medicine, spectroscopy, archeology, transportation, food, and security [76–87].

### CNNs in classification: architecture and components

An example of a CNN model and its components is given at the bottom of Figure 3. The different components are explained below.

#### Input layer

The input layer contains the images. In supervised classification tasks, the defining labels (also called ground truth) are supplied with the images. Together, these create the data set that will be used to train the model and evaluate it.

#### Convolutional layer

**Figure 4:**
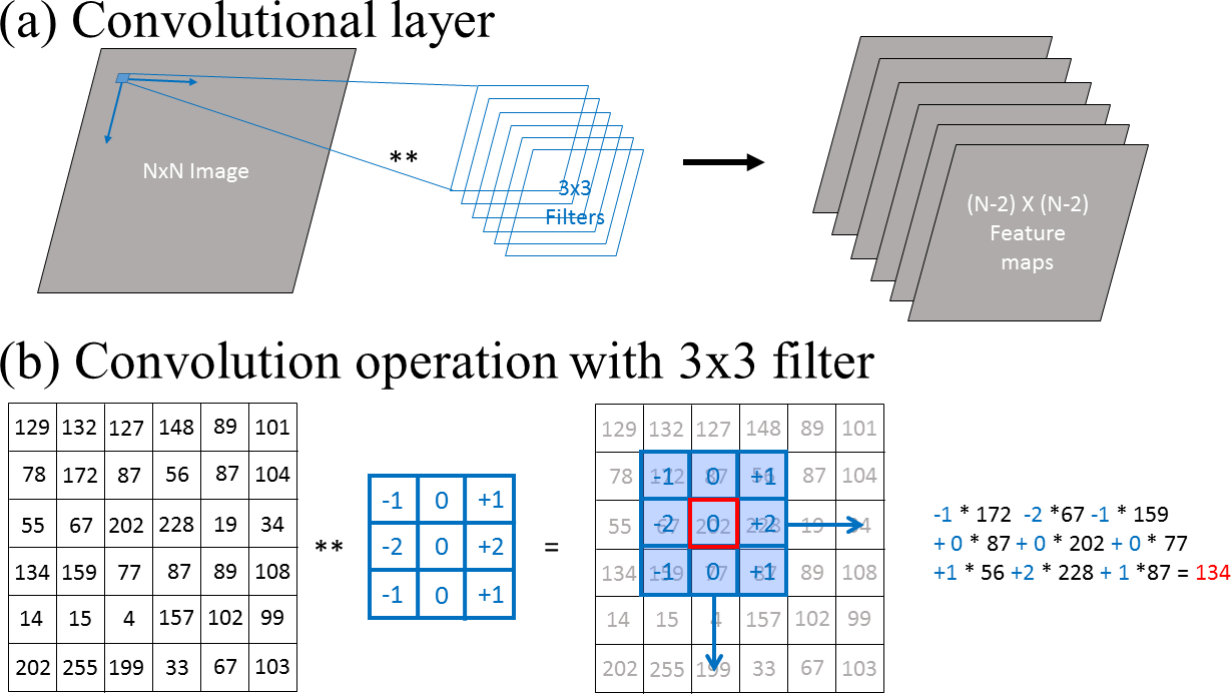
(a) Description of the convolutional layer of a CNN and (b) the convolution operation. In (b) the output of the convolution operation by the 3 × 3 filter at a given position of the image replaces the value of the central pixel at this filter position (red cell). The convolution operation is applied on the whole image with a fixed stride (number of cells the filter is moved between operations. A common stride is 1 in both *x*- and *y*-dimensions).

A convolutional layer (Figure 4), is a layer of filters (i.e., kernels). Each filter is an array that contains parameters. Each filter is convoluted with each image resulting in mappings of the extracted features. These are called “feature maps”. The number of feature maps for an image is the number of filters applied to the image. In standard image processing techniques, kernel filters exist for functions such as edge detection, edge enhancement, noise reduction, and smoothing. In contrast, in CNNs, the parameters of the filters are not known a priori and the CNN model learns the best parameters during the training process to extract the features that best differentiate between images from different categories. Dimensions of the filters for 2D convolution typically have dimensions of 3 × 3, 5 × 5, and 7 × 7.

#### Activation layer

An activation is usually applied on the feature maps to give “activated” feature maps. This is typically a non-linear operation that produces the non-linear decision boundaries. Without activation, the output would be that of the kernel operation itself, so no new information would be gained in the propagation. The activation emphasizes or de-emphasizes the importance of the different output kernel operation values relative to the others. A very popular activation function is called rectified linear unit (ReLU), which returns the input value for positive numbers and 0 for negative numbers. Other common non-linear activation functions are the sigmoid, tanh (hyperbolic tangent) and Leaky ReLU, the difference between them being how the input (to the activation) is transformed. The choice of activation function affects both the learning efficiency and the learning decision boundary. ReLU, in general, is more efficient and effective than the sigmoid and tanh activations in CNNs and facilitates the learning of more accurate and sophisticated decision boundaries. However, the optimal activation function is task-dependent.

#### Pooling layer

The pooling layer is used for down-sampling the extracted features in the feature maps and is usually applied after the activation layer. It compresses the feature maps while keeping the most important information of the features. Usually, a window of size 2 × 2 (for 2D convolution) slides on a feature map in strides of 2 × 2 and the maximum value within the 2 × 2 pooling window is returned. This kind of pooling is called “Max pooling”. The convolutional, activation, and pooling layers together form an operation block. Usually, this block is applied several times. Each block corresponds to a hidden layer. The number of filters can be increased or remain the same in each successive block, and the feature maps are down-sampled leading to dimension reduction while keeping the most important information.

#### Flatten layer

In the last block there are many small feature maps for every image that passed through the CNN. The output of the last block is flattened (and hence termed flatten layer) to form a vector of numbers. For example, if the output of the last block is 128 feature maps of size 6 × 6 each, then each feature map is flattened to a column vector of size 36 × 1 (6*6). Finally, all these vectors (128 vectors, each of size 36 × 1) are stacked vertically together to form a column vector of size 4608 × 1 (36*128). The flatten layer enables the use of linear algebraic operations in further mathematic processing. This is followed by a dense layer, that is, a “fully connected layer” that connects the flattened output to subsequent neurons. There may be several such dense + activation layers that create the classifier as shown in Figure 3 (bottom).

#### Output layer

The output layer is the last layer of the CNN that contains “neurons”. Here, the number of neurons is the number of image categories in the image classification task (in binary classification tasks, one “neuron” is sufficient instead of two “neurons” (see below). The activation function of this layer is chosen by the operator to reflect the given task.

#### Activation functions for the output layer

In this step, a number between 0 and 1 is assigned for each image corresponding to the probability that it belongs to a specific category. This is performed by applying an activation function (e.g., sigmoid for binary classification or softmax for “multiclass” systems, that is, systems that have more than two classes into which images can be classified). The softmax function returns values between 0 and 1, similar to the sigmoid function, so that each class in a given image is assigned a probability. The summation of the probabilities for all classes in a given image is equal to 1, and the class with highest probability is the most probable class prediction. In binary classification, probability values close to 0 indicate that the image is likely to belong to the first category, while values close to 1 indicate a higher probability that the image belongs to the second category and usually a classifier probability threshold of 0.5 is chosen to differentiate between the classes.

#### Loss function

The above output, together with the true labels of the images are used for calculating the error in the predictions (the loss function). The loss function is optimized (by an optimizer/algorithm) to yield the lowest error. In each iteration, images are fed to the CNN, and the loss at output is calculated. This process is called forward propagation. Then, the model parameters (internal parameters of the model that are learned in the training process) of the CNN are updated to yield lower loss. This process of updating the parameters is called backward propagation. The gradient of this function with respect to the parameters then indicates whether the changes are indeed reducing the loss toward a minimum. The process of forward and backward propagation is done many times until some accuracy/loss threshold is reached. The loss function has different formulae for different tasks. For example, binary-cross entropy, or categorical-cross entropy losses can be applied, respectively, for binary and categorical classification, while for regression problems, appropriate losses could be the mean squared error or mean absolute error losses [28,88].

#### The problem of over-fitting

When the model performance on the training set is much better than the performance on the testing set, the model is over-fit. Specifically, decision making is strongly influenced by random noise and meaningless trends in the training data. This boosts performance on the training dataset, but results in poor generalization to new data. There are several approaches that can reduce over-fitting.

**Increasing the sample data set** size helps reduce over-fitting. Since there are more examples in the data set, a more general model is obtained. Collecting more data would increase the data set size, but this may not be feasible. Another solution is data augmentation.

**Data augmentation** [9–10,13] is a method to increase the sample data set size, for example by a set of transformations such as rotations, shape distortions, color distortions, flipping, or change of scale. This method is “free of charge”, that is, it provides more usable data from the existing set, and can increase the sample data set size by several times.

**Using less complex models with less parameters:** As with any model, deep learning models can be fit better by increasing the number of parameters, but more parameters lead to over-fitting [26,28]. Using a less complex model with less parameters (less hidden layers and less neurons) will reduce over-fitting.

**Using regularization methods:** “Regularization is any modiﬁcation we make to a learning algorithm that is intended to reduce its generalization error but not its training error“ [28]. A common regularization method in deep learning is called dropout [31,89]. Dropout randomly shuts down some of the “neurons”. Consequently, in each training iteration, some of the “neurons” are ignored and their parameters are not updated during the optimization. This leads to reduction in the memorization of the training set resulting in better generalization. Dropout layers [13,18] in CNNs are usually used after the dense layers. Additional regularization methods apply penalties on the layer parameters, generally, making their absolute value smaller. The process of shrinking the parameter values is known to increase the generalization of the fit to new unseen data which in turn leads to less over-fitting.

**Transfer learning** is applying the knowledge gained from one problem to a different but related one [90]. For small data sets, one can use models that already trained on large data sets and then tune only some of the parameters, usually the last layers. In that case the models that trained on the large data set learned important low- and high-level features from the larger data set, and tweaking of the parameters is done by freezing the parameters from the first layers and tuning the parameters of the last layers.

**Tuning hyperparameters of the model with *k*-fold cross-validation:** A validation set is used for several tasks in model development. One of these tasks is tuning of hyperparameters. The hyperparameters are external parameters of the model that are tuned, for instance, the number of neurons/kernels or kernel size, while the parameters of the models are internal, and learned automatically from the data (i.e., weights and biases). In this procedure, the model is trained on the training data for a given set of hyperparameters and then for each set of hyperparameters the model performance is tested on the validation set (a subset of the data set aside for this purpose). The hyperparameters that yield the best results on the validation set are chosen for the final model. The performance on the validation set should reflect the true performance that will be obtained for real unseen data, but in some cases it gives an overly optimistic result. In *k*-fold cross-validation the data set is split *k*-fold and trained *k* times on a *k* − 1-fold set. Each time, the model uses the remaining fold as a validation set for estimation. The *k* validation sets are used for model tuning to obtain the optimal model before testing. This enables tuning the model with respect to *k* validation sets and reduces the associated over-fitting by finding the optimal set of hyperparameters of the model with respect to *k* validation sets.

**Figure 5:**
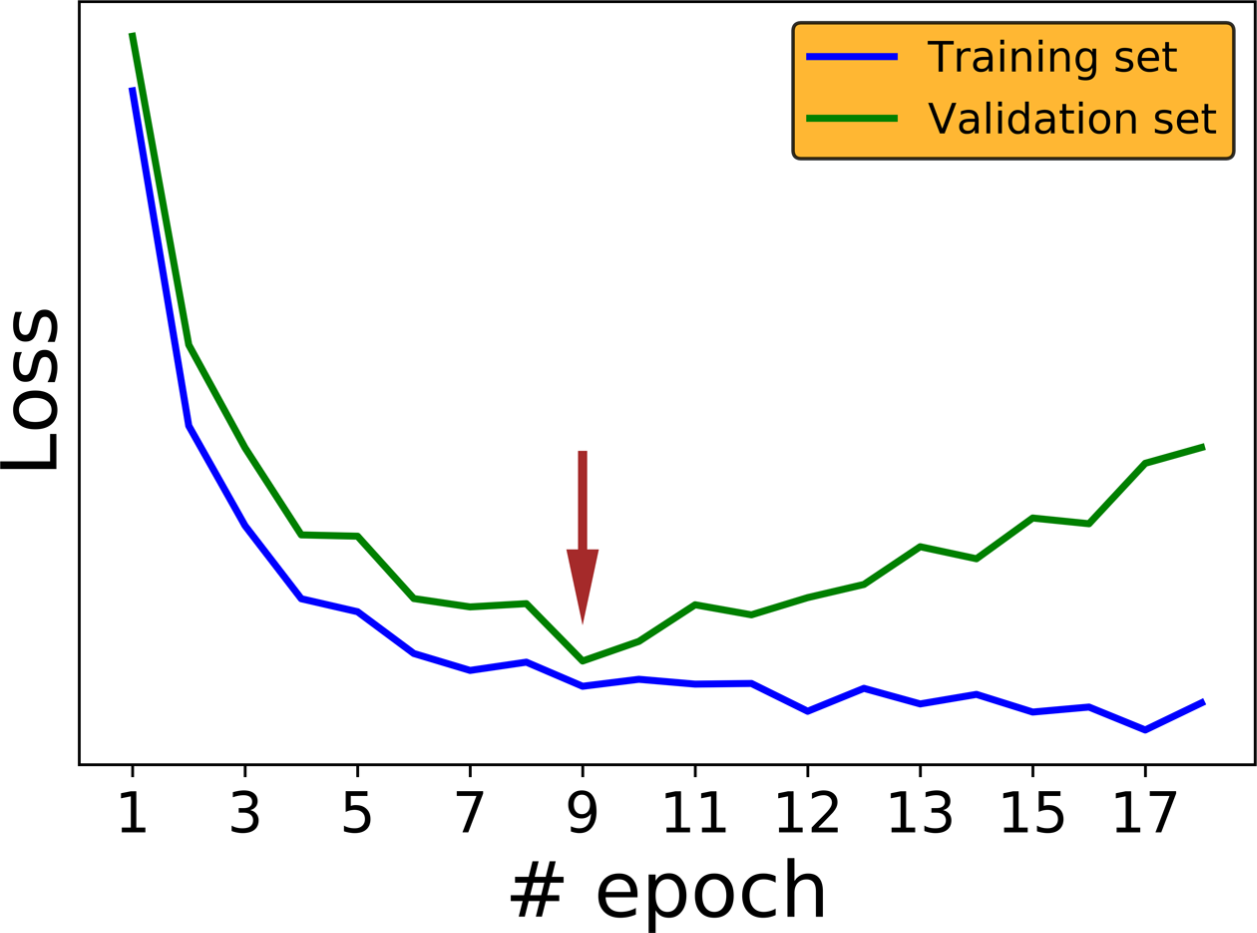
Loss function as a function of the number of epochs for the training set (blue line) and the testing set (green line). The brown arrow demonstrates the stage when early stopping should be applied.

**Early stopping of the training process:** One way to prevent over-fitting is to stop the training process before it over-fits. In that case the model does not memorize the training set and better generalization can be achieved. The loss functions of the training and validation sets decrease for successive epochs (learning cycle of entire data set) but eventually the loss of the validation set starts to increase while the loss of the training set continues to decrease. This crossover is the point where the training is terminated (Figure 5).

### Deep learning in microscopy

The profound impact of deep learning on the field of microscopy was emphasized in a recent editorial in “Nature Methods”: “In microscopy, we would be hard pressed to identify a trend that has so captured the current zeitgeist as deep learning. Not since the introduction of super-resolution microscopy methods over a decade ago has a class of tools had such potential to disrupt microscopy as we know it” [91]. In this section we will describe the use of DNNs in general and CNNs in particular in the field of microscopy [7,9,33,92–94], beginning with the more abundant examples of optical and electron microscopy, which will later be compared and contrasted with applications in SPM [95].

#### Strategies for obtaining training data

Successful application of deep learning relies on the quality (e.g. signal/noise, resolution, and contrast) of the training data. Obtaining good training data can be very challenging, which is one of the limiting factors of this method. Collecting a sufficient number of images can be complicated and time-consuming [7]. There are several methods to acquire training data. One of the common ways is to experimentally record the necessary sets of both high- and low-quality images, depending on the application. For example, de Haan et al. obtained pairs of low-and high-resolution SEM images by changing the magnification of the images [96]. Weigert et al. acquired pairs of low- and high-quality images by varying the exposure and laser power of the confocal microscopy images [97]. Pairs of healthy and diseased leaf photographs from different plants were collected in order to develop a deep learning model for disease detection [97–98].

Acquiring sets of images can be a complicated and prolonged task, especially for the relatively slow scanning probe microscopy as discussed below. Acquired optical images are significantly influenced by the recording conditions such as illumination, focus, and noise. In cases of low quality or limited number of experimental images it is possible to apply computational approaches to improve them, for example by enhancing contrast and increasing the amount of training data by data-augmentation methods mentioned above [7,97], or by using classical image reconstruction methods such as nearest neighbor, bilinear, or bicubic interpolations [99].

One way to exploit the power of computation to circumvent difficulties in sufficient data acquisition is to train deep convolutional neural networks on simulated data. Deep STORM is a deep convolutional neural network that achieves high-resolution images from images recorded by a standard inverted microscope under bad imaging conditions, such as low signal-to-noise (S/N) ratio or high emitter (photon) density, leading to blurry images. The network can be trained either on experimental or on simulated images. Simulations are based on the well-understood physics of the image degradation process to generate training images. The simulated videos approximately matched the experimental parameters for tracking submicrometer-scale particles in videos recorded from a light microscope [100–101].

A generative adversarial network (GAN) [102] comprises two adversarial networks, namely a generator network, which generates fake images that look like real images and a discriminator network that learns to discriminate between the fake and true images. The two networks compete with each other and are trained alternately: initially, the generator simulates images that are very different from real ones, then the discriminator learns to discriminate between the fake and true images. With training, the discriminator improves, and the generator learns to generate fake images that look real. This process repeats itself until the discriminator cannot distinguish between the fake images generated and the real ones [103–104]. GANs have been used to achieve segmentation without human annotation [104]. In this approach, computer-generated images are used for segmenting true bright-field images by synthesizing colorful segmented images and then overlaying them on the experimental image. In this way, successful segmentation of a wide variety of image types was achieved, such as in images of cells and worms, enabling efficient cell-counting and viability prediction, as well as of silver nanowires.

#### Image classification and segmentation

CNNs are popular for cell classification. A CNN model based on actin-labeled fluorescence microscopy images detected differences in the actin cytoskeleton structures between cancerous and normal breast cells that were not discerned by the human eye [105]. Nitta et al. developed an intelligent real-time cell sorter, by combining deep-learning with microfluidics. They achieved automated classification of continuous high-speed and blur-free bright-field and two-color fluorescence image acquisition of cells flowing at a rate of 1 m/s [106].

An outstanding CNN for image segmentation is U-Net, developed mainly for biomedical image segmentation [107]. It leads to precise and fast segmentation of images and requires relatively few images to train compared to other deep learning applications. U-Net has been applied successfully in many applications of image segmentation. Another openly available segmentation tool, termed CDeep3M is cloud-based and the CNN functions are in a plug-and-play format. This tool is widely applicable for different image segmentation tasks encompassing both 2D and 3D image types. The authors demonstrated segmentation of membranes, mitochondria, and nuclei for images of a mouse brain slice using different microscopic techniques such as CT X-ray microscopy, electron tomography, fluorescence microscopy, and serial block-face scanning electron microscopy (SBEM) micrographs [108].

Deep learning techniques have been developed for segmentation and tracing of neurons in volume electron microscopy for synaptic connectivity reconstruction. Mitochondria, synapses, axons, dendrites, spines, myelin, somata, and different neuronal cell types were automatically identified [109].

Li et al. developed a tool, AutoSiM, for automatic classification and segmentation of single-molecule fluorescence time traces through deep learning [110]. In this approach, a recurrent neural network (RNN, see [28]) was applied, which is appropriate for applications involving sequential data (such as time series). The application in this work is classifying and segmenting time traces with respect to the ground truth, that is, the presence or absence of a probe binding to a mutant sequence. The authors also applied an alternative approach to the RNN for classifying two-channel time series data by applying a CNN. Here, the data from each trace (donor and acceptor intensity values) was converted into a 2D scatter plot used as an input image for the CNN, thus converting the RNN classification task into a CNN classification task. AutoSiM was shown to yield results nearly equivalent to manual selection in much shorter time.

Masubuchi et al. developed a deep learning-based, autonomous robotic system for exfoliated 2D material detection in a motorized optical microscope. Efficient detection of various exfoliated 2D crystals (van der Waals heterostructures, graphene, hBN, MoS_2_, and WTe_2_) was demonstrated. The detection algorithm enables real-time detection of the 2D materials (running for 200 ms on a 1024 × 1024 optical image) and is insensitive to variations in microscopy conditions such as illumination and color balance. This is a fundamental step in the development of automated fabrication systems for these materials [111].

#### Object tracking

Objects are tracked by following them in a movie or a series of time-lapse images. Reliable object tracking depends on the segmentation accuracy [7]. Moen et al. applied deep learning and linear programming (an optimization method) for tracking single cells in live-cell imaging of both fluorescent and bright-field images of the cell cytoplasm [112]. Newby et al. developed a CNN for fully automated submicrometer-scale localization of particles such as viruses, proteins, and drug particles from image data. A diverse collection of video conditions (different signal-to-noise ratios, background intensity, frame size, particle size, and motion) was used to generate the training data. The neural network tracker showed good accuracy for both 2D and 3D simulated videos and 2D experimental light microscopy videos [100]. Also here, the particle path linking was done by applying linear programming to construct the tracking task. Recently, efficient object tracking has been achieved using CNNs only [113]. These approaches carry promise for improvements in object tracking tasks [114].

#### Image processing

**Augmented microscopy:** A common practice in microscopy is to attach a fluorescent label to components of interest in order to identify them unequivocally. In addition to the additional workload this requires, such labeling can alter properties and interactions of the species under investigation. Furthermore, this method will only reveal labeled components. Those that were not or cannot be labeled will not be highlighted. Low-cost label-free microscopy can be computationally augmented to add pseudofluorescent cellular labels to the image [115]. By using deep learning frameworks, fluorescent labels representative of cell type, state, or specific cellular component (organelles) can be identified and selected from non-labeled transmitted-light microscopy images. Christiansen et al. developed a computational machine learning approach, which they call ‘‘in silico labeling’’ (ISL), that can predict fluorescent labels in transmitted-light images of unlabeled biological samples. ISL can predict labels for nuclei, cell type, and cell state [116].

**Image restoration and de-noising:** In fluorescence microscopy, imaging speed, spatial resolution, light exposure, and imaging depth are all limited by the optics of the microscope, the fluorophore chemistry, and the photon exposure tolerance of the sample. In order to mitigate effects of the latter, Weigert et al. developed an image restoration deep learning network that enhances the range of observable biological phenomena by recognizing images acquired with 60-fold fewer photons. The network was trained with pairs of images with low S/N ratio as input, and images with high S/N ratio as the target output images [97]. By training a GAN to transform diffraction-limited input images into super-resolved ones, Wang et al. improved the resolution of wide-field images acquired with low-numerical-aperture objectives to resemble those acquired using high-numerical-aperture objectives. The method was also applied to confocal microscopy images and total internal reflection fluorescence (TIRF) microscopy [117].

The use of CNNs for improving image quality and temporal resolution for super-resolution localization microscopy (such as PALM and STORM) has been widely investigated [103,118]. For example, Ouyang et al. presented ANNA-PALM, a deep learning approach for image reconstruction. Super-resolution images are generated from sparse, rapidly acquired photo-activated localization microscopy and wide-field images. High-quality, super-resolution images of microtubules, nuclear pores, and mitochondria can be reconstructed from low-resolution images with two orders of magnitude fewer frames than usual. This shortens acquisition time and reduces sample irradiation [119].

Deep learning was also applied to reduce noise and increase the resolution in scanning electron microscopy images. An approach for creating a training dataset of low- and high-resolution image pairs from a single image, was developed by using deep learning to up-sample scanning transmission electron microscopy (STEM) images after nearest neighbor down-sampling. This enabled an increase in image resolution of up to 100-fold, decreasing scanning time and electron dose [120]. Another application of CNNs for STEM was for atomic defect classification [121]. The goal was to characterize defects related to Si scattered on a graphene surface. In this work two different CNNs were applied, first a larger scale “sniffer” to locate areas in the atomic lattice displaying irregularities and then an “atom finder” to characterize the unique chemical arrangement near the defect found in first network. The second network was trained on simulated STEM images. Then, scanning tunneling microscopy (STM) images of the same sample were used to characterize the defects. STM images, which give the local density of states, measure not only the Si lattice, but also defect areas where this well-ordered lattice disappears. Such images were compared with those computed by density functional theory (DFT) based on well-known single and dimer Si defects.

The examples given here demonstrate the utility of deep learning in general and CNN in particular in the field of microscopy. In the following section, the emphasis is narrowed from general microscopy to scanning probe microscopy (SPM), which provides unique opportunities to exploit the power of CNNs.

### CNNs applied to SPM

Whereas CNNs have proven themselves extremely useful for a variety of microscopic images, scanning probe microscopy (SPM) images have qualities that make the approach for this application unique. First of all, conventional SPM images typically take much longer to acquire than optical or electron microscope images. This requires devising means to generate enough images for both the training and the test set. In some cases, particularly when analyzing single molecules, the test set can be generated computationally from first principles. Various fast-scanning approaches have also been applied. Augmentation techniques are often required. Another characteristic of SPM is that it is multimodal. One scan can provide multiple mappings simultaneously with the topography, for example, adhesion, phase shift, stiffness, work function, or friction. In the following section, the utility of CNN in SPM is illustrated through several examples taken from the literature.

#### Enhancing speed of image acquisition

As discussed above, SPM imaging is inherently slow. One of the ways to improve speed is to reduce the number of pixels and scan lines that need to be measured. For instance, presuming constant time per pixel, a 256 × 256 pixels image will take four times longer to image than a 128 × 128 pixels image. Such a reduction in pixels will naturally reduce the image resolution, and may not only be aesthetically less satisfying, but possibly result in failure to observe important surface features. One of the deep learning methods to enhance resolution is called very deep super-resolution (VDSR, very deep due to large number of convolutional layers, in this case 20). This method was applied to low-resolution AFM images, resulting directly in higher resolution results [122]. The main obstacle to effective implementation of this method for AFM studies is the dissimilarity of different samples, making the choice of training sets critical. In this case, two sets, consisting of high-resolution AFM images, were used, that is “monomaterial” consisting of one ceramic sample, and “multimaterial” consisting of multiple samples of more complex nature (e.g., shell, bone, or nanoparticles). These were taken at various resolutions. The multimaterial set of images was six times larger than the monomaterial set. High-resolution images were then computed in a test set starting from a low resolution image of 32 × 32 pixels, resulting in “high resolution” images of 64 × 64, 128 × 128, and 256 × 256. These were directly compared to the AFM images taken at these higher resolutions. The images indeed were able to recoup some of the hidden features, but seem to provide only very marginal improvement over simple bicubic interpolation.

Luo et al. used a method called μ-scanning where, instead of scanning each pixel, full and consecutive scan lines are replaced by random line segments comprising only parts of selected scan lines [123]. Thus, much fewer pixels need to be measured. Since the tip needs to be retracted from the surface, moved to a new place, then re-engaged for each such segment, the placement and arrangement of these line segments represent a trade-off between longer segments, which are more time-efficient, and shorter ones that should enhance fidelity due to greater randomness. The deep architecture included two networks, one to generate the overall image, and the other to retrieve the fine features, with 15 layers between the two networks. In principle, the biggest room for deviation from reality is the reproduction of features extending across several scan lines, which could be missed by random gaps of several lines in the corresponding line segments. The training process applied transfer learning, which used an existing library of animal images (which are insensitive to scale). The deep CNN was shown to give better performance in much less computing time than alternative, filling methods. The resulting CNN images gave better fidelity compared with full raster scans made 25 times faster than a standard raster that provided the “ground truth” image, 70 s as opposed to 30 min. The μ-scan also took 70 s.

The onset of high-speed AFM (HS-AFM) has created additional opportunities but also challenges for researchers that could be aided by machine learning [124–127]. The challenges in HS-AFM are twofold: 1) How to ensure that sufficient data can be collected in a short period of time, and 2) how to reliably extract information from the vast number of images that are created? Machine learning techniques, in general, can be used to increase the image acquisition speed, by reducing the number of data points that need to be acquired, as discussed earlier. In data-driven control strategies such as the ones developed in one of our labs [128] the traditional optimization-based solvers can be replaced by machine learning, thereby ensuring faster and more accurate tracking of the sample without exciting unwanted resonances.

CNNs are particularly promising for automating the post-processing of the HS-AFM images. AFM images need to be post-processed to remove unwanted image features such as background slopes and image artifacts. The large number of images can be best handled by automation of this process. Conventional methods for automated image processing of HS-AFM data based on algorithms containing specific assumptions about the sample are often not robust when used for arbitrary samples [129–131]. CNNs work well in noise removal and could help in background subtraction, scar removal, and detection of other imaging artifacts. A recent review highlights the efficiency of various CNNs for noise removal compared to conventional de-noising [132].

Extracting quantitative information about the dynamics occurring in the sample from fully processed HS-AFM images is a time-consuming task, since one experiment can consist of tens of thousands of images. Hence, the bottleneck can shift from data acquisition to data processing. Here machine learning in general and CNNs in particular can efficiently and quickly process the images to identify and quantify dynamic processes. The complexity of such analysis is much greater than when the CNN extracts information from one image. In tracking applications, information from HS-AFM images also needs to be correlated between images in order to provide the temporal information, for example as is needed for the extraction of rate constants. This is further discussed below in the discussion on object tracking.

On the topic of enhancing imaging speed, we briefly mention a publication which, although it did not make use of a CNN, used machine learning to distinguish properties of AFM images. Here, the authors sought an automated way to distinguish between healthy and cancerous cells. Thus, images were acquired using an enhanced scanning technique termed “ringing mode”, which simultaneously gives mappings of other surface properties together with topography [133]. In this case, it was found that whereas topography was a poor criterion for separating the two populations, adhesion was much better. Furthermore, the images themselves were not the input to the machine learning, but rather feature engineering was applied to determine relevant global parameters from the image analysis. These included, among others, roughness as average or root mean square (RMS), surface skewness, surface kurtosis, and peak–peak. This approach greatly reduces the dimensionality of the data space. Three different machine learning methods were applied, two of them unsupervised. A random subset of the parameters was input to the architecture and it was trained to divide into healthy or cancerous cells.

#### Applications in multimodal and non-imaging techniques

As mentioned at the beginning of this section, one of the strengths of SPM is that it is multimodal, that is, several different physical phenomena are measured in one scan. This was exemplified by application of a CNN to extract physical information under low S/N conditions for band-excitation piezoresponse force microscopy (PFM) [134]. Band excitation collects a band of frequencies around the contact resonance frequency of the tip–sample system, which is modeled by a simple harmonic oscillator equation. This allows for the determination of several physical quantities, that is, frequency (giving the stiffness), amplitude (giving the piezoresponse), *Q* (dissipation), and phase (directionality of polarization). PFM can map piezoelectric domains and the inverse piezoresponse of a sample, but signals are notoriously low. In this work, two arrays (real and imaginary parts of the single harmonic oscillator data) were fed into the network. It was shown that the DNN outperforms the traditional least-squares analysis when S/N is low, but at the cost of accuracy. However, further optimization can improve this situation. Feeding the DNN result into the least squares analysis gave the best result. The authors suggest that using data obtained before from neural network analysis as input to model fitting could be extended to other modalities of SPM, such as magnetic force microscopy and Kelvin probe force microscopy.

Another recent example of application to a non-topographical SPM technique is the study of ferroelectric switching [135]. This switching is a function of both reading and writing voltages, and can vary with experimental conditions such as time and temperature, and is further complicated by competing processes. The measurement technique was contact KPFM, which has an inherently low signal due to damped response of the tip in contact with surface. In this case, the data is not surface mappings, but rather hysteresis loops in graphical format, which were “unfolded” by plotting the voltage response as function of the read voltage and writing voltage step number, rather than the actual voltage value (which would result in a loop). This had the result of producing characteristic mappings rather than a series of overlapping loops, the latter having proven intractable to interpret. Converting the spectral data to image format facilitates its treatment by standard image analysis techniques such as interpolation in order to tweak out more information. Different types of switching were clearly grouped by principle components to capture changes in switching behavior at different temperatures and different curve types. A neural network was applied to these images. The advantage over the algorithms applied earlier was that nonlinear boundaries could be set between the different clusters of behavior. This network succeeded in identifying the ferroelectric behavior from the maps at different temperatures for temperature differences as small as 5 °C.

A shallow neural network was used in an attempt to eliminate topographical artifacts contributing to the signal in scanning thermal microscopy. In this technique, the probe is essentially a thermocouple. Such a probe is relatively bulky, and on a rough surface will be in proximity to a locus of surface points. The neural network treatment was compared to finite element analysis and a simple estimation of the sampled volume using blind reconstruction. Finite element analysis gave a significantly better result than the NN but was very slow. The NN was much faster, but did not perform significantly better than the blind reconstruction [136].

A common means to distinguish between healthy and sick cells is the elastic modulus, determined by measuring the stiffness with AFM force–distance curves and then fitting to a model. Several works have applied machine learning to analysis of the force curves. The data here is not images, and CNNs were not used, but this will be briefly presented as an important and growing use of deep learning for AFM. Neural networks were applied to distinguish between force curves of healthy and cancerous cells without human intervention, which is traditionally carried out by fitting curves to a model to obtain a value for modulus [137–138]. In this case, rather than using the model-derived value of elastic modulus, the classification was done by curve shape, without explicitly producing a modulus value. Machine learning enabled identification of artefactual force curves relative to good curves [139–140].

#### Segmentation object recognition, and tracking

Microscopy images, AFM being no exception, are sometimes challenging to segment. This is because the different classes that need to be segmented could be overlapping or touching, and sometimes too similar to one another. Furthermore, noise and other artifacts in the image can be mistaken as an object and uneven levelling makes it impossible to threshold features at a particular height. It was recently shown that the CNN U-Net, introduced above, could be used to classify different types of agglomerations of nanoparticles in AFM images [141]. U-Net was chosen because it works well on relatively small training sets (in this case 428 images, which was also a manageable size for the supervised learning applied here), and the fact that it provides precise localization, as required for segmenting such features. This is accomplished through a “U-shaped” architecture, whereby the feature maps derived from the images are first contracted (through convolutions and pooling) then expanded through convolutions and up-sampling to associate the important features with a specific location. The network was compared with two other common segmentation methods, namely thresholding on a local mean value and Otsu's threshold, which optimizes the variation in pixel values between the final segments produced. The different methods were compared not only for success in segmentation, but also for invariance in pixel assignments when noise and artifacts were added to the images. In this work, although U-Net did not consistently outperform the more standard segmentation methods, it was more generally applicable, as Otsu's threshold and local mean are each optimal for (different) specific image types.

In a parallel approach to this problem, the same researchers showed that segmentation for aggregates of nanoparticles is tractable without manual training [142]. In this case, a larger training set of 2500 images was generated by Monte Carlo simulations. The different regimes of aggregation (cells, labyrinth, particles, holes, and liquid) could be generated based on different starting conditions of the Monte Carlo run, so that the simulated images were automatically labeled. The training features were generated at different size scales (scans from 500 nm to 90 μm size) to make the CNN scale-invariant. Furthermore, an “autoencoder” was applied to remove noise. This was trained on images to which speckle noise was added to learn how to remove it. Without removing the noise, the results are unsatisfactory. The CNN network used was based on a classifier developed by the Oxford Visual Geometry Group (VGG), and had only two hidden layers. A large kernel size of 32 × 32 and 3 × 3 strides was used in the first layer and a kernel size of 64 × 64 was used in the second layer. This approach succeeded in correctly classifying two thirds of the images as compared to less than 10% obtained by random selection. The classification will run into trouble when different regime types appear in one image. By subsampling, it was possible to locate such situations. 83% of them were correctly located and discarded, and 74% of the images expressing only one regime were passed. Clearly, this classification could not be used as a stand-alone method, but could be used to filter through large numbers of images to find likely candidates that could be further evaluated by other means.

Yablon and Chakraborty used a CNN to distinguish between AFM phase images of two different kinds of polymer blends. In this case, they had only 160 images for training and testing. Nonetheless, the CNN was able to make the distinction with 100% accuracy relative to a simple neural network, which had 94% accuracy [143].

Banterle et al. [144] used the machine learning software Ilastik [145] on PORT-HS-AFM images [146] to study the self-assembly kinetics of the centriolar protein SAS-6 into ring-shaped cartwheels. The authors trained Ilastik to detect 14 different classes of homodimer assemblies. With this classification, they then calculated the probability for each object in an image to be of one of the 14 classes. The time evolution of each of these objects was then fitted for each PORT HS-AFM movie to the coagulation–fragmentation equations of this system. A global fit of the time-dependent curves of the 14 species resulted in estimations for the values of *k*_on_ and *k*_off_ for the SAS-6 cartwheel self-assembly process.

A CNN was recently used to track and characterize the self-assembly of proteins on a surface [147]. In this study, the self-organization of DHR10-mica18 proteins adsorbed on mica was studied using HS-AFM. In general, ordered molecules on a surface yield better-resolved images than disordered ones. Thus, the final image of the time series, consisting of assembled and ordered proteins, was used as ground truth with well-ordered and resolved protein molecules. These were labeled manually, then the training set was prepared by various augmentation methods (e.g., random cropping, zoom, and scan-line streaks). This was fed to a neural network to classify each pixel as either belonging to background or to a protein “particle”, then fitting each object to an ellipse. In this way, the position and orientation of each molecule in each frame was obtained. This definitive identification enabled the application of further complex analysis to define the trajectories of the molecules.

The increased imaging speed of HS-AFM also facilitates the use of AFM as a manipulation tool. CNNs can play a crucial role in automating processes and ensure robust nanomanipulation. For example, Bai et al. [148] have used an instance segmentation network and a fully convolutional network to detect movable nanowires in an AFM image. This is a required step toward achieving autonomous AFM nanomanipulation-based assembly of complex structures.

The capability of scanning probe microscopy to image molecules at high resolution has converged with modern computational techniques to enable insight into molecular structure and properties. Two works employed CNNs to examine single-molecule images, one using STM and the other using AFM. Kalinin et al. used a CNN to determine the up/down orientation of sumanene molecules on a crystalline Au surface [149]. Analyzing the molecular images is complicated by the fact that each molecule can adsorb to the surface in four different azimuthal rotational angles, and furthermore can be blurred by the STM tip, which provides enough energy to cause molecular rotations. 25,000 images were generated for training using DFT simulations of the different rotational classes and a Monte Carlo sampler. The specific state including up/down and rotation was determined through a combined Markov mean field for determining the up/down orientation and CNN/principle components analysis [150] for determining rotation. This was possible even for cases where the molecule was between one of the four rotational states.

Alldritt et al. also examined highly resolved images of molecules that could absorb in various orientations on a surface, making their identification difficult and somewhat subjective [151]. High-resolution AFM images can provide information complementary to that of STM. The ability to measure forces while probing molecules allows for the highly-resolved reproduction of the force field between the CO molecule adsorbed on the tip and the molecule of interest adsorbed on the surface. Deflections of this CO probe define the 3D data mapped by the probe, represented by “slices” made by scanning at different heights. Considering the complexity of such images, such as the fact that the force interaction is quite short range so that displacing the tip upwards by atomic dimensions will cause features to disappear, human interpretation is impossible without computational intervention. Transforming these force slices into a molecular model, known as a reverse-imaging problem, is highly complex and non-linear. A CNN was applied to learn this inverse function from sample atomic structures and corresponding AFM data. Such maps take hours to acquire experimentally. Therefore, this work also used a DFT-generated training set of over 100,000 molecules. In its present state, this model cannot fully deal with small molecular perturbations due to tip and/or surface interactions (which is work in progress). Nonetheless, the CNN could quickly and efficiently return accurate 3D reconstructions of complex molecules presenting different orientations on the surface from both experimental and simulated images.

#### Characterization of scanning artifacts and tip distortions

The use of feature engineering in DNNs has recently been applied to characterize the extent of sample distortion caused by the tip [152]. Surfaces were artificially generated with different degrees and natures of roughness, which were characterized by several parameters such as rms roughness, skewness, kurtosis, and lateral correlations. Simulated scans were performed on such surfaces using model tips to generate certainty maps showing where the tip actually encountered the surface. Such certainty maps are needed for the process of blind reconstruction, where the contribution of the tip shape to the acquired image is reproduced.

When imaging well-defined objects such as single molecules, it is possible to use the quality of such images and the shape of repeated image features to determine the condition of the tip. This is well known in AFM and STM work and commonly used to characterize the tip shape [153]. In high-resolution STM, procedures have been developed to “condition” a damaged tip and restore its sharpness. To connect between these two processes, imaging and conditioning, Rashidi et al. applied a CNN in a form of feedback so that when the image quality indicates that the tip is damaged, the tip conditioning routine, consisting of pushing the tip into the surface, is initiated [154]. This routine is cycled continuously with additional imaging and CNN analysis to tell the operator when the tip conditioning has been successful and it is possible to continue imaging. In this work, the training set consists of 2800 STM images of dangling bonds on a hydrogen-terminated Si(100) surface (augmented eight times by image transformations). These images were classified manually. From a larger image, then, the dangling bonds are identified as bright spots and squares of 28 × 28 pixels containing these features represent the data that are fed into the CNN. There are two possible classifications, namely single or double tip. The CNN was compared to five other image-classifying models, including a fully connected neural network, and performed significantly better with 97% accuracy. In an example given, a double tip was identified, and four conditioning cycles were interspersed with imaging until results indicated that a sharp tip was obtained. This method still required some user intervention to choose the proper area for imaging and conditioning. Nonetheless, it is an important step toward achieving autonomous atomic-scale fabrication.

In a sense, single molecules and atomic defects are an ideal test case due to their relative ease of calculation for training and the predictability of the different types of artifacts and permutations that could appear. Whereas the above work considered only two types of tips (sharp and double), Gordon et al. included several other types of common tip artifacts. This work considered both features that would normally be assigned to the tip (e.g., double tip) as well as those associated to the surface (e.g., steps). In addition to H-terminated Si, they also studied Au and Cu single-crystal surfaces [155]. Expanding the set of samples allowed for the inclusion of more general artifacts. For instance, dimer and row artifacts are characteristic of Si but would not necessarily appear on other surfaces. The single-crystal surfaces included features such as step edges and impurities. This work, like the previous, required manual acquisition and then classification of the images, including not only different tip states, but also artifacts appearing on the surface, such as contaminants and step edges. Images were classified as good or bad, depending on whether or not there were certain tip and surface artifacts. The images thus are quite specific to the type of surface studied, and implementation requires massive human input (both imaging and classifying). The authors subsequently developed a method to assess the tip information with only a few scan lines, potentially providing for a gain of two orders of magnitude in CNN speed [155–156]. But the fundamental issue of massive supervision requirement and the need to characterize each surface type needs to be overcome to make this more widely appealing.

A proposed solution to parts of the bottleneck in applying CNNs for automated SPM shows that it is possible to develop more autonomous operation, in this case, for STM imaging of MgPc on Ag [157]. The goal here was completely autonomous SPM, demonstrated by 86 h of unaided scanning, during which scanning areas were determined to be suitable or not, images were acquired, damaged tips were fixed, and new scanning areas were chosen. The code for this procedure is available from the authors. Although an initial training was still required (7600 64 × 64 pixels images were manually acquired and classified as good or bad), all other steps did not require human intervention. A tip was determined to be bad after the acquisition of four consecutive bad images. It was then moved to an appropriate surface region for conditioning. Using reinforcement learning, the tip conditioning process was optimized by having the tip undergo one of twelve different operations (either voltage pulse at different voltages or pushing the tip into surface to different depths) and the result of the actions was rated on awards basis. This allowed for the independent optimization of the method. The authors state that this could be applied to any sample or even different SPM applications, such as spectroscopic or lithographic ones, but such extensions would apparently still require labor-intensive acquisition and classification of training sets.

A final example has nothing to do with image analysis or AFM operation, but rather with aesthetics. For microscopic techniques such as SPM, the choice of color applied does not have a true physical meaning but is used for aesthetic and contrast purposes. The coloring is defined by luminosity, and two color channels, namely “A” for the red-to-green spectrum and “B” for the blue-to-yellow spectrum. A recent work used a CNN to color greyscale images [158]. This work identifies “semantic features” (well-defined and characteristic features on the image). Then these are compared to a reference figure for colorization, which distinguishes the different semantic features with different colors. This is done in four stages. The first stage identifies the semantic features in the data set and is called an encoder. In an intermediate stage, finer information is extracted with a model using transfer learning and combined with the encoded data with a convolutional layer. Finally, a network called a decoder assigns the colors. The NST-CNN trains on a reference model, then uses the weights to color the microscopy images. In fact, the images used here are SEM images but the principles used in this demonstration are proposed for application to AFM.

### Detailed example: a CNN for classifying AFM images of red blood cell cytoskeleton

In this final section, we give a detailed example of the application of CNN for classification of AFM images recently applied in our lab [67]. We attempt to provide here a detailed, step-by-step description of the CNN application. The task was to provide an objective measure of the morphological differences of cytoskeleton images known to be from healthy and from damaged red blood cells. Specifically, HS-AFM images of the cytoskeleton of healthy cells (“Healthy”) were compared to images from cells exposed to extracellular vesicles (EVs) derived from *Plasmodium falciparum* (malaria)-infected cells (“*Pf*-EV exposed”). The AFM images were used together with independent biochemical methods to monitor the damage to the cytoskeleton. Due to the inherent biological variability between samples and the inhomogeneity of the cytoskeleton substructures, images from damaged cells could not be easily distinguished from images of healthy ones. Two expert scanning probe microscopists achieved, on average, 75% success in distinguishing the images of the different sets.

CNN models were then developed to classify the cytoskeleton images of healthy and *Pf*-EV exposed cells. After affirming the validity of the analysis, we applied the model to an experiment in which the malaria-derived EVs were pre-treated with the proteasome inhibitor Bortozomib to inhibit the cellular damage (“Treated”). Such treated cells should be immune to the damage, and indeed the CNN found their images to be similar to those of healthy cells. The CNN approach applied in this work is similar to approaches developed in recent years for biomedical and texture deep learning image applications [41,44,105,159–169].

#### The data sets

The two image sets obtained with the HS-AFM, batch 1 and batch 2, correspond to two experiments performed on different days; the cells in each set therefore came from different donors, underwent slightly different washing procedures and were measured with different AFM tips. The first set comprised 60 healthy cytoskeleton images and 65 *Pf*-EV exposed cytoskeleton images, and the second set comprised 51 healthy cytoskeleton images and 58 *Pf-*EV exposed cytoskeleton images. 2 μm x 2 μm images were acquired with 512 × 512 pixels resolution.

#### Development of the CNN models

The different CNN architectures were tuned first, reserving 20% of the images as a test set. The remaining 80% of the 512 × 512 pixels images were further randomly grouped into 20 different sets (for model architecture and hyperparameter tuning), 75% for training and 25% for validation. Each image was divided into four non-overlapping images of 256 × 256 pixels size. Four rotations of 0°, 90°, 180°, and 270°, as well as horizontal and vertical flipping augmentation was performed on the training set. The resulting training sets were trained to give an accuracy of 96% or for 30 epochs, whichever occurred first. After training the model, we evaluated the accuracy using the validation sets. Here, instead of applying early stopping with respect to the validation sets as in Figure 5, we preferred to train the CNN until it reached optimal performance on the training sets while tuning the model architecture on the validation sets to yield optimal performance, since the validation losses stabilized for these data sets under these conditions. Accuracy is defined as the number of images classified correctly divided by the total number of images.

We trained our models on a 7th generation Intel Core™ processor, with four cores, eight threads and processor base frequency of 2.8 GHz. Training in parallel 2400 images of size 256 × 256 pixels each, on all eight threads, takes 30–35 s per epoch with a batch size of 24 images, and 15–17 min for 30 epochs. The CNN architecture giving the best average accuracy on the validation sets is shown in Figure 3 (bottom). Table 1 describes the different CNN architectures that were examined.

**Table 1:** Description of the different examined CNN architectures.

	Number of convolutional layers	Number of filters in each layer	Number of fully connected layers	Number of neurons in the fully connected layer

architecture 1 (Best)	5	(8, 16, 16, 32, 64)	1	256
architecture 2	4	(8, 16, 16, 32)	1	256
architecture 3	5	(16, 32, 64, 64, 128)	1	512
architecture 4	5	(32, 32, 64, 64, 128)	1	512
architecture 5	5	(32, 64, 64, 64, 128)	1	512

To further improve the CNN performance, we applied architecture 1 again on a combination of training and validation sets, with the above described image augmentation transformations. This protocol, which resulted here in more than 2000 images for each set, has been suggested as an approach to reduce over-fitting [31].

#### Performance on testing

**Figure 6:**
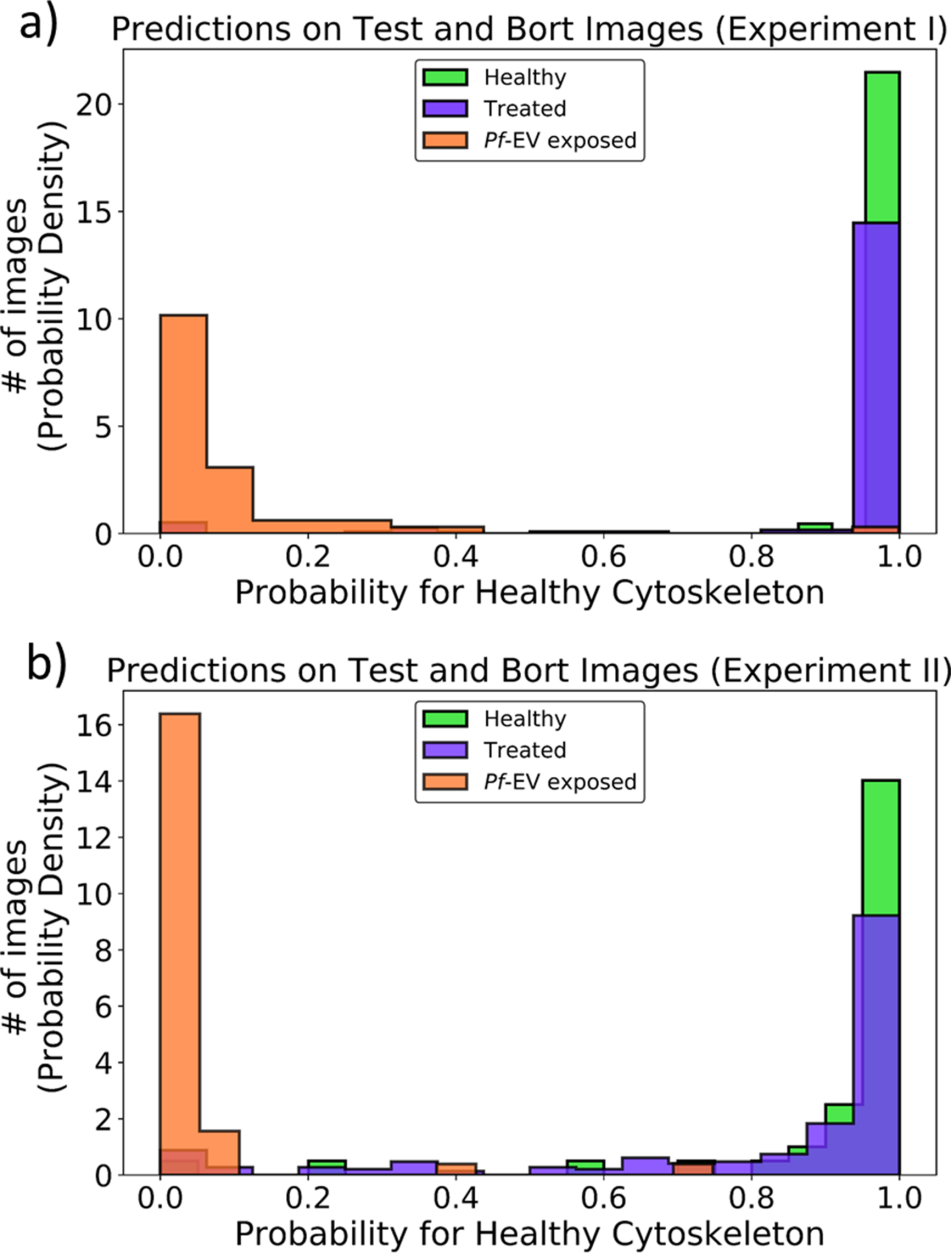
Predictions of the probability for a healthy cytoskeleton made by the model on testing set and treated samples cytoskeleton images from (a) data set 1 and (b) data set 2. The *x*-axis is the probability and the *y*-axis is the number of images in probability density units (100 “Healthy” and “*Pf*-EV exposed” images and 188 “Treated” images for data set 1, and 88 “Healthy“ and “*Pf*-EV exposed” images and 236 “Treated” images for data set 2). “Healthy” (green bins) are the images of healthy cytoskeleton, “*Pf*-EV exposed” (orange bins) are the images of cytoskeleton of cells exposed to malaria-derived EVs, and “Treated” (purple bins) are the images of treated cytoskeleton. The figures show that the model is successful in distinguishing between healthy, untreated, and *Pf*-EV-treated red blood cells.

With this model we obtained accuracies of 99.0% and 96.6%, respectively, for testing data sets 1 and 2. The results for these test sets are shown in Figure 6. Since these two data sets come from different experiments, they formally belong to two different experiments, that is, cells from different sources, prepared on different days, and measured with different probes. A generalized model for the combined data, trained on all the images from both sets was performed. This training required a more complex model to capture the variety of the additional image features and resulted in a somewhat lower accuracy (86.1%). We ascribe the lower accuracy to some overfitting due to the relatively small number of images available. Nonetheless, this is significantly better than what was achieved by the two human SPM experts.

In order to demonstrate the advantage of the CNN over traditional machine learning algorithms, we applied random forest modeling on the data sets. First we flattened each image in the data sets into a 1D vector, where each pixel is a feature in that vector. Then we applied fivefold cross-validation on the training set together with a grid search on the number of estimators. The best models yielded accuracies of 86.0% and 67.0%, respectively, on the testing sets of data set 1 (with 350 estimators) and data set 2 (the number of estimators in that case depended on the randomness of the algorithm, and different runs gave different number of estimators for best model performances, while the accuracies were similar in all cases). These accuracies are significantly lower than those obtained with the CNN, and show the power of CNN for image applications.

#### Obtaining further insight

As an independent test of this model, we applied it to the Bortozomib-treated samples. As mentioned above, this treatment is designed to inhibit *Pf*-EV-induced damage, and treated cells should be similar to healthy ones, Application of the model used above for the individual sets predicts 94.1% of the 188 images in batch 1 and 86.0% of the 236 images in batch 2 to be healthy. These results are shown in Figure 6.

**Figure 7:**
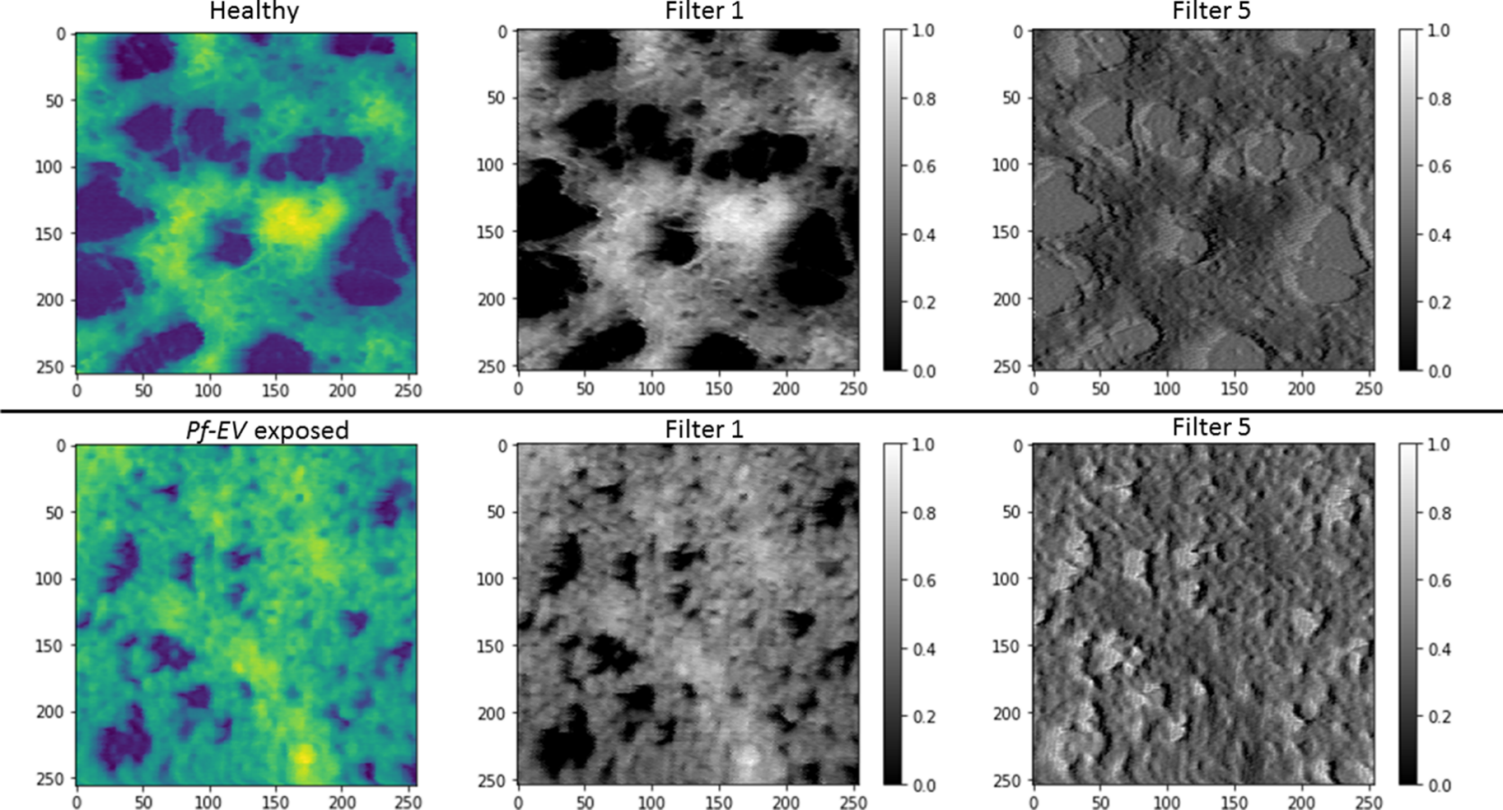
Example of images of healthy (top) and diseased (bottom) *Pf*-EV exposed cells from the test set and the output of filters 1 and 5 of the CNN, that is, the feature maps. The features associated with the filters are brighter, as indicated in the color bar. The feature map intensities were normalized to 0–1. xy scales are in pixels, with each pixel equivalent to 4 nm.

**Figure 8:**
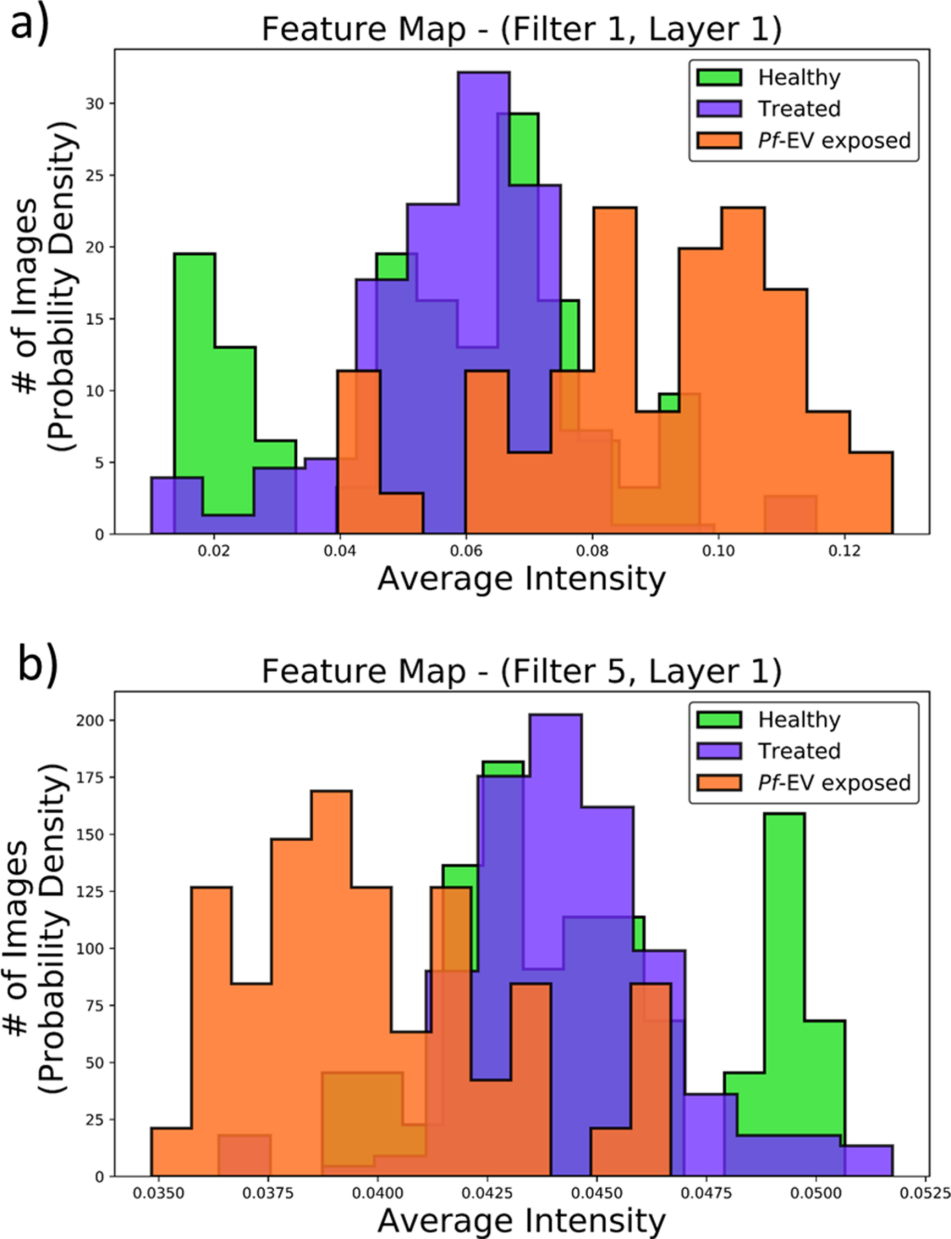
Average intensity for all images in the testing set and the images of treated cytoskeleton for (a) feature map 1 in layer 1 for data set 1 (backbone) and (b) feature map 5 in layer 1 of the CNN (holes). Green: images of healthy cytoskeleton, (purple) images of treated cytoskeleton, and orange images of *Pf*-EV-exposed cytoskeleton.

Having established that the Bortozomib treatment significantly preserves the cell cytoskeleton structure, the CNN model is applied to the following questions: 1. What are the main features that differentiate between healthy and *Pf*-EV-exposed cells? 2. How similar are treated and *Pf*-EV-exposed cells to healthy cells? To answer these questions we exploit information found in the feature maps [32] of the first CNN layer (low-level features). In data set 1, we observe that feature maps 1 and 5 (Figure 7), represent backbone and holes of the cytoskeleton, respectively. For each image, the intensities of these feature maps were averaged, and the distribution of these intensities is shown in Figure 8.

**Figure 9:**
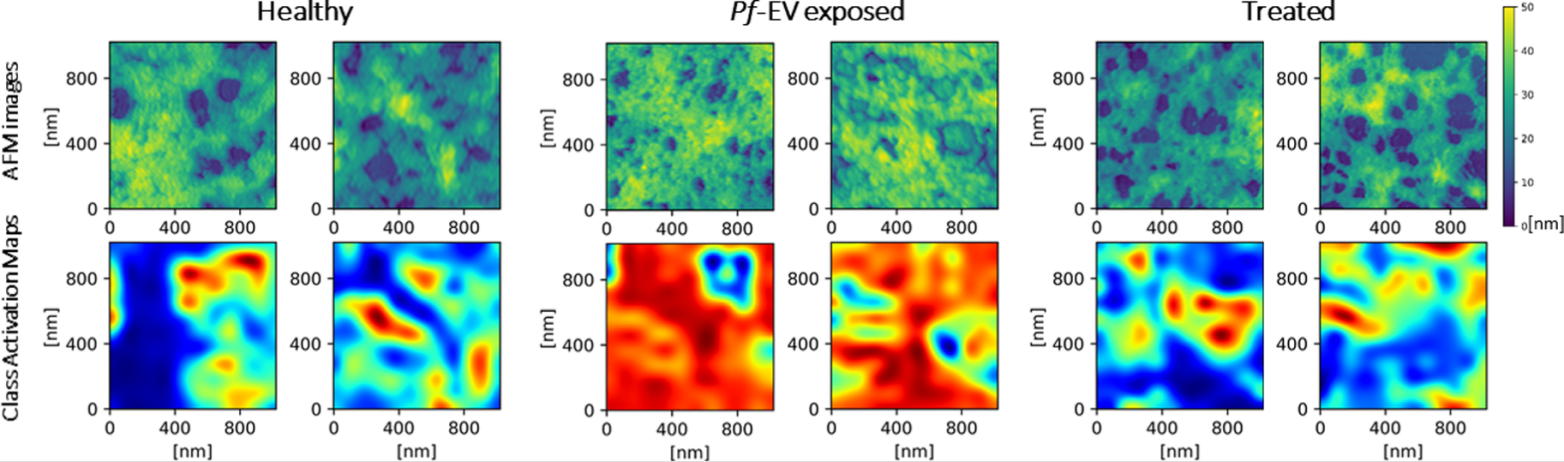
Class activation maps shown below the AFM images for selected images of healthy and *Pf*-EV-exposed cells from the test set and of treated cells from data set 1. Red/yellow regions are discriminative regions for a given class, while the blue regions either have no effect on the class or are anti-correlated.

These distributions show that feature map 1 (backbone) is statistically more relevant for the *Pf*-EV-exposed cells than for the healthy and treated cells, and the opposite is true for feature map 5 (holes). This implies that in the *Pf*-EV-exposed cells, there is more backbone than holes and in the healthy and treated cells there are either more holes or that the holes are larger. Structurally, this must be true because holes come at the expense of the (digested) backbone and vice versa. In this sense, the CNN has located these segments on the images. Next, to show the discriminative image regions used by the CNN to identify the image class, we calculated the class activation maps for selected images in data set 1 [170–171]. In this approach, features from the final feature activation maps (last CNN layer) are weighted according to their importance and summed to give the class activation map. Then the class activation map is up-sampled to the size of the input image. The procedure described in [170] was implemented in the following way: starting with the trained model and replacing the last pooling layer with the global average 2D pooling layer, in other words, each feature map is reduced to one representative number. Then this output (vector of size 1 × 64) is connected to two output units for the two classes with softmax activation. We fine-tuned only the weights of the last layer that connects the global average 2D pooling output layer to the two class output units. The results for selected images from the testing set of data set 1 are shown in Figure 9. Discriminative regions of the class appear in red/yellow and regions with no importance or that are anti-correlated with the class are blue. This clearly demonstrates that the holes are correlated with the healthy and treated cells and serve as the discriminative regions while the backbone is not correlated or anti-correlated with that class. The opposite holds for the *Pf*-EV-exposed cells. By this we answer question 1: In data set 1, the backbone is more dominant than holes in *Pf*-EV-exposed cells. In healthy and treated cells, the holes are more dominant. It is important to emphasize that the analysis demonstrated in Figure 7 and Figure 8 is only a low-level distinction. Further, more complex features and relations are discerned in the subsequent layers, but these are less physically tractable in this case.

To answer question 2, we applied Welche’s *t*-test on the different populations presented in Figure 8. We found a significant difference between healthy and *Pf*-EV-exposed populations with very small *p* values (below 10^−10^) and a non-significant difference between healthy and treated populations with much higher *p* values (above 0.2). By this, we answer question 2 and quantify the similarity between the healthy and treated cells.

**Figure 10:**
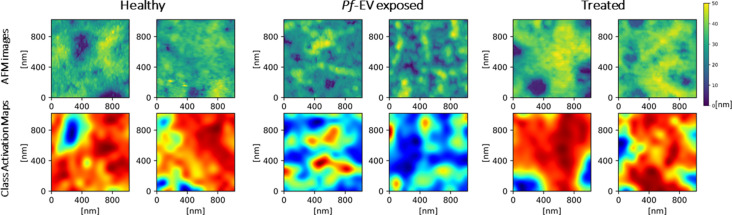
Class activation maps shown below the AFM images for selected images of healthy and *Pf*-EV-exposed cells from the test set and of treated cells from data set 2. Red/yellow regions are discriminative regions for a given class, while the blue regions either have no effect on the class or are anti-correlated.

For data set 2, class activation maps obtained for selected images are shown in Figure 10. The analysis reveals that the discriminative features for that data set are somewhat different from data set 1 and thus experiment-dependent. Specifically, the discriminative regions for the healthy and treated cells are large “connected” backbone regions while the discriminative regions for the *Pf*-EV-exposed cells are smaller “discrete” backbone regions.

The code for this work was written in Python v. 3.6.8. In particular, for machine learning and deep learning implementations the packages Scikit-learn v. 0.21.1 [172] and Keras v. 2.2.2 [173] were utilized. In addition, the Scipy v. 1.2.1 package for data analysis [174] and the OpenCV v. 3.4.2 package for computer vision [175] were used for specific tasks.

## Conclusion

Applications of CNNs to SPM measurements and their analysis is a relatively new, but exciting field full of potential. This review has highlighted the utility of this approach in identification and classification of objects ranging from single molecules to defect sites on ordered lattices, through larger domains and complex subcellular structures. In addition, applications in lithography, manipulation, and spectroscopy have been considered. As computational techniques improve and fast scanning becomes more widespread, the reliability of these approaches will increase significantly.

Applications of CNNs are already proving to enable new, exciting science and technology. Writing atomic-scale circuit components represents the ultimate in data storage density [176]. However, the writing speed is unrealistically slow for commercial use. Automating the writing of atomic-scale binary information autonomously, as shown above in [154], could be an important factor in pushing this lab-demonstration into a technologically relevant tool. The reinforcement learning applied in this example, as well as in [157], are excellent examples of enabling the automation of scanning probe work, an area poised to become more prevalent as the methods become more efficient and accessible. An excellent review of reinforcement learning can be found in [73].

It should also be noted here that, in addition to the traditional role in instrument control, human input is a critical part of the data interpretation. The ability of machine learning to process enormous data sets can give rise to new hypotheses and interpretations that could have been neglected or proven intractable without machine learning. This is especially relevant to SPM where the multimodal character adds significant complexity to data interpretation, providing new opportunities when this complexity can be exploited properly. The use of feature engineering can significantly simplify the multidimensional aspect of SPM imaging. The last section shows that even with a relatively small number of images resulting from SPM experiments, an efficient and reliable CNN model can be formed. Furthermore, class activation maps can be used to determine the significant features in the images. As indicated throughout this review, the generality of the models and libraries used, and their rapid and wide development in many fields, can be expected to accelerate the developments in this budding area.

Whereas the aim of this review was to highlight the remarkable achievements as well as the potential of CNNs for applications in SPM, we have also included some examples using traditional machine learning or general neural network applications. Machine learning has a longer history of application in SPM work. Some earlier examples can be found in [177–178]; it is also used in some commercial SPM applications. The combination of CNNs with traditional machine learning methods such as principle components analysis, Bayesian optimization, and others was exploited in several of the works discussed here and will likely remain an essential strategy. Overall, one can say that this field is not just a classic example of experimental advances pushing computational ones, and vice versa, but rather a paradigm change in scanning probe microscopy.
